# Supplementary material for: DMRforPairs: identifying Differentially Methylated Regions between unique samples using array based methylation profiles
Source: BMC Bioinformatics. 2014 May 15;15:141. doi: 10.1186/1471-2105-15-141 (PMC4046028; doi:10.1186/1471-2105-15-141)
Supplement: Additional file 2 — DMRforPairs output for the comparison of A431-MCF7 and NA17018-NA17105. Please start from the HTML files in each folder. Available via the BMC Bioinformatics website. [file 1471-2105-15-141-S2.zip › 1394847754114233_MOESM2_ESM/A431_MCF7/figures/18563.pdf]

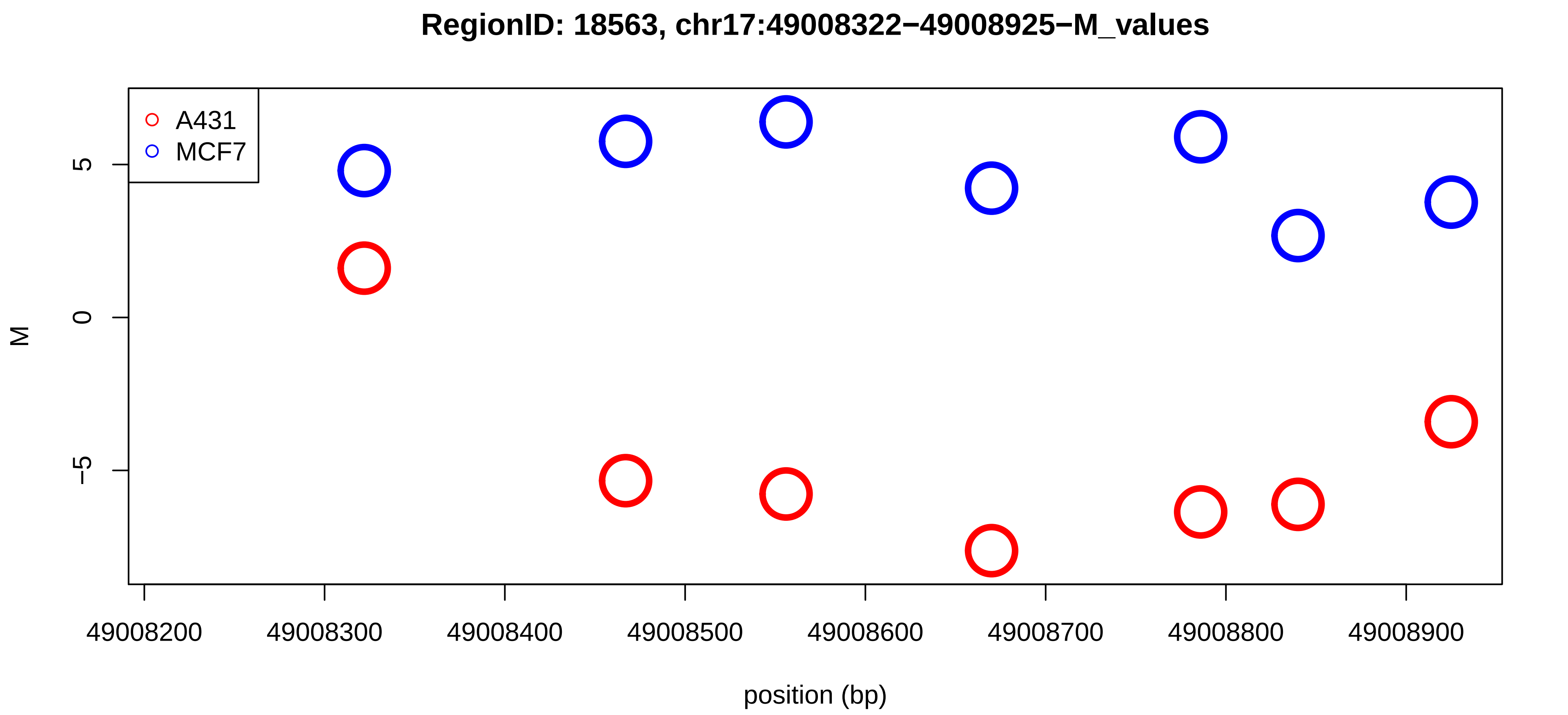

RegionID: 18563, chr17:49008322−49008925−M_values
A431
MCF7
M
5
0
−5
49008200
49008300
49008400
49008500
49008600
49008700
49008800
49008900
position (bp)

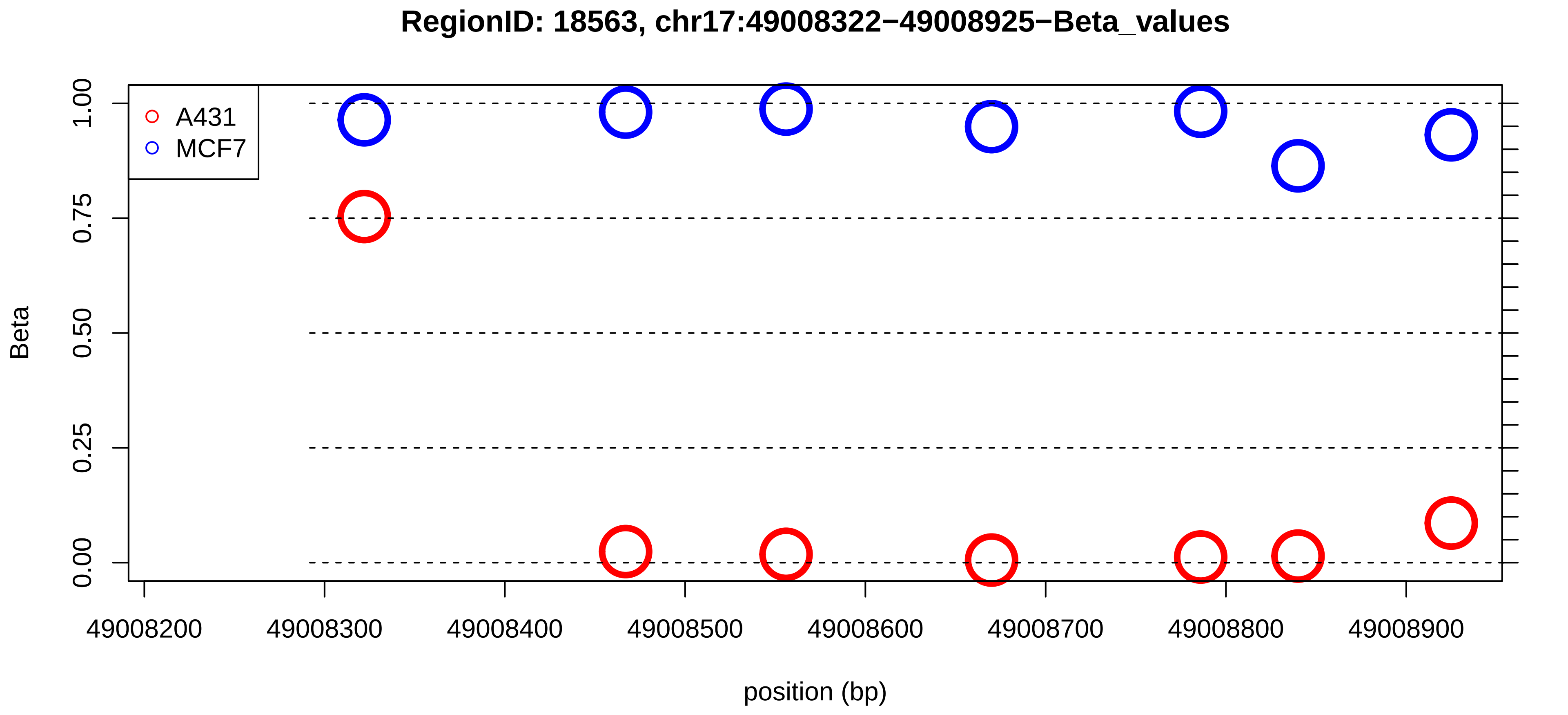

RegionID: 18563, chr17:49008322−49008925−Beta_values
A431
MCF7
Beta
1.00
0.75
0.50
0.25
0.00
49008200
49008300
49008400
49008500
49008600
49008700
49008800
49008900
position (bp)

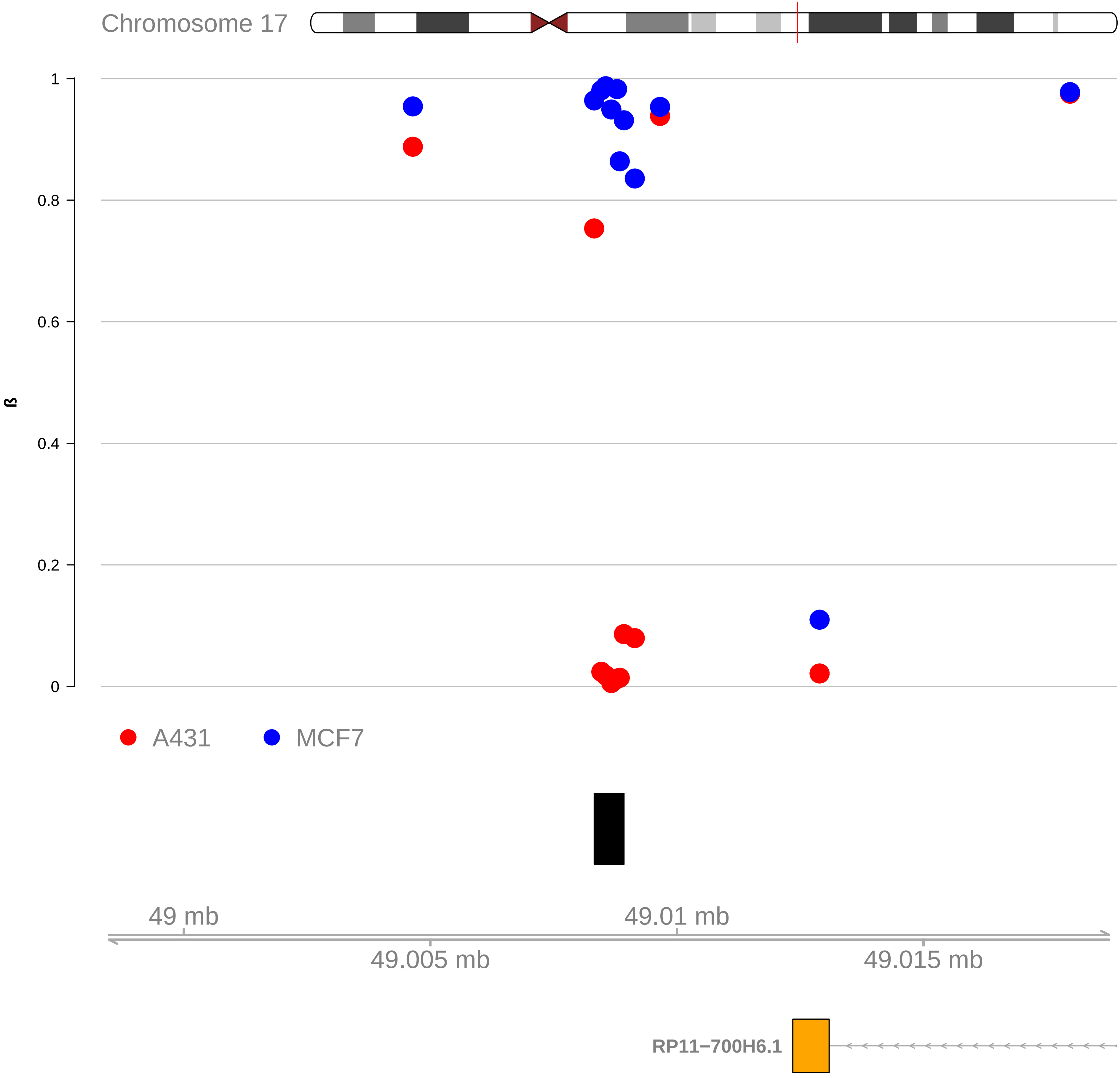
Chromosome 17
1
0.8
0.6
0.4
0.2
0
ß
A431
MCF7
49 mb
49.005 mb
49.01 mb
49.015 mb
RP11–700H6.1
